# Image-guided, Minimally Invasive Evacuation of Intracerebral Hematoma: A Matched Cohort Study Comparing the Endoscopic and Tubular Exoscopic Systems

**DOI:** 10.7759/cureus.3569

**Published:** 2018-11-10

**Authors:** Cristoph Griessenauer, Caroline Medin, Oded Goren, Clemens M Schirmer

**Affiliations:** 1 Neurosurgery, Geisinger Health System, Danville, USA; 2 Neurosurgery, Geisinger Health System, Danville , USA

**Keywords:** hemorrhage, hematoma, intracranial evacuation, minimally invasive surgery, endoscope, exoscope, tubular retractor

## Abstract

Introduction

Novel image-guided, minimally invasive techniques to evacuate intracerebral hematomas represent a promising new avenue in the management of this disease entity. To our knowledge, a direct comparison of the Penumbra Apollo (Penumbra Inc, Alameda, California, US) and Nico BrainPath (Indianapolis, Indiana, US) system has not yet been performed.

Methods

A retrospective review of image-guided, minimally invasive evacuation of intracerebral hematomas performed at one academic institution in the United States between July 2015 and July 2017 was performed. Cases performed with the Apollo and BrainPath system were matched based on age, gender, hematoma location and laterality, and volume.

Results

Twenty-four patients underwent surgery using either of the two minimally invasive surgical systems and five cases in each group were matched for age, gender, hematoma location and laterality, and volume. Median time from symptom onset to evacuation was two days with a mean distance from the brain surface to the clot of approximately 40 millimeters in both groups. Both techniques achieved comparable clot evacuation. The functional outcome was poor with either technique with the majority of patients dependent or dead at last follow-up.

Conclusions

In the present, small, matched cohort study, both the Apollo and BrainPath techniques achieved satisfactory clot evacuation. Nevertheless, the functional outcome in this patient population remains poor in the majority of cases.

## Introduction

Nontraumatic intracranial hemorrhages (ICHs) account for 10%-15% of all strokes and have a devastating natural history [1]. The primary injury from ICH is characterized by mechanical destruction and ischemia of the surrounding brain tissue. Risk factor modification and prevention are the only avenues aimed at this phase. The subsequent release of blood products and proteins from the hematoma heralds the second phase of post-ICH injury and represents a target for therapeutic intervention. Whereas traditional surgical strategies have failed to demonstrate a benefit for hematoma evacuation [2-3], promising results from the Minimally Invasive Surgery and Thrombolysis for ICH Evacuation (MISTIE) phase II trial [4] as well as for the development of other image-guided, minimally invasive evacuation techniques, such as the endoscope-assisted Apollo (Penumbra, Alameda, CA, US) and exoscope-assisted BrainPath (Nico Corporation, Indianapolis, Indiana, US) systems have revived hopes for successful surgical ICH treatment. Non-prespecified subgroup analyses from trials such as MISTIE II suggest a possibility that minimally invasive evacuation compared to open surgical surgery benefits some ICH patients, but there are no data to compare minimally invasive endoscopic and tubular retractor systems. In the present study, we performed a matched cohort study comparing the Apollo and BrainPath systems aiming to address the question of whether the modality of minimal evacuation plays a role.

## Materials and methods

Data collection and matching

Approval for this study was obtained from the institutional review board. As all information was obtained retrospectively, the need to obtain patient consent was waived. A retrospective review of image-guided, minimally invasive ICH evacuations using either the Apollo or the BrainPath system between July 2015 and July 2017 at one academic institution in the United States was performed. Patient demographics, ICH characteristics (i.e. location, laterality, volume, associated intraventricular hemorrhage (IVH), and etiology), clinical presentation (i.e. Glasgow coma scale (GCS) upon admission), treatment parameters (i.e. time from onset to evacuation, postoperative volume), and outcome (i.e. GCS upon discharge, length of stay, modified Rankin score (mRS) on last follow-up) were recorded. Pre- and postoperative hematoma volume was calculated on OsiriX (Pixmeo SARL, Bernex, Switzerland) by performing regions of interest segmentation of the hematoma and volume calculation. Cases performed with the Apollo and BrainPath systems were matched in a stepwise fashion based on age, gender, hematoma location and laterality, and volume.

Operative techniques

Both Apollo and BrainPath ICH evacuations were performed in the operating room under general anesthesia using Brainlab (Munich, Germany) neuronavigation. The technical aspects of both procedures have been previously described in detail [5-6]. In short, for Apollo cases, a 19 French sheath was inserted through a burr hole or mini-craniotomy with less than two-centimeter diameter to the distal aspect of the hematoma along its longest axis using neuronavigation. The Apollo wand was inserted through the working channel of the LOTTA endoscope (Karl Storz, Tuttlingen, Germany) and blood product pushing into the sheath was aspirated under direct visualization and with copious irrigation from distal to proximal. At the end of the procedure, the Apollo wand, the endoscope, and the sheath were removed. For BrainPath cases, a small craniotomy was made after a suitable trajectory had been identified. The dura was opened in a slit or cruciate fashion over a sulcus and the arachnoid over the sulcus was opened using the standard microsurgical technique. Using the transsulcal approach, the BrainPath port was introduced along the planned trajectory and the obturator removed. A Vitom exoscope (Karl Storz, Tuttlingen, Germany) was centered over the port using the nitrogen-powered zero-gravity pneumatic holder Point Setter (Mitaka Kohki Co., Tokyo, Japan). Using standard bimanual techniques, the clot was removed using standard surgical suction and, in some cases, using an automated, non-ablative resection device Myriad (Nico Corporation, Indianapolis, Indiana, US).

Statistical analysis

The statistical analysis was performed with JMP statistical software (SAS, Cary, North Carolina, US). Ordinal values were compared using Pearson’s test. P-values of < 0.05 were considered significant.

## Results

**Figure 1:**
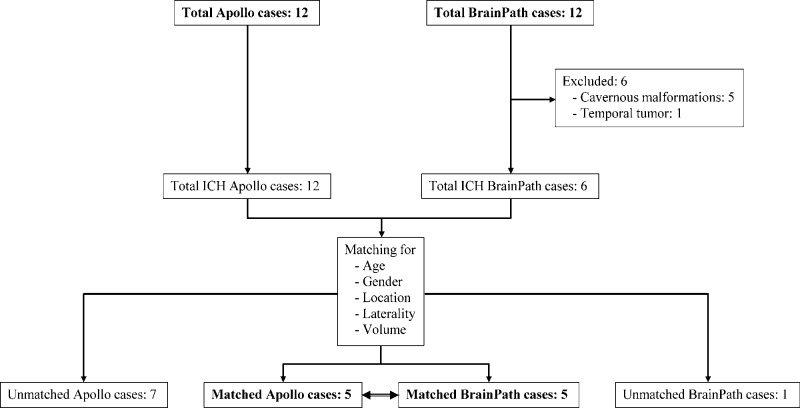
Enrollment and matching diagram ICH: Intracranial Hemorrhage

Twenty-four patients underwent surgery using either of the two minimally invasive surgical systems. This included 12 hematomas evacuated using Apollo and seven using BrainPath (Figure 1).

**Figure 2:**
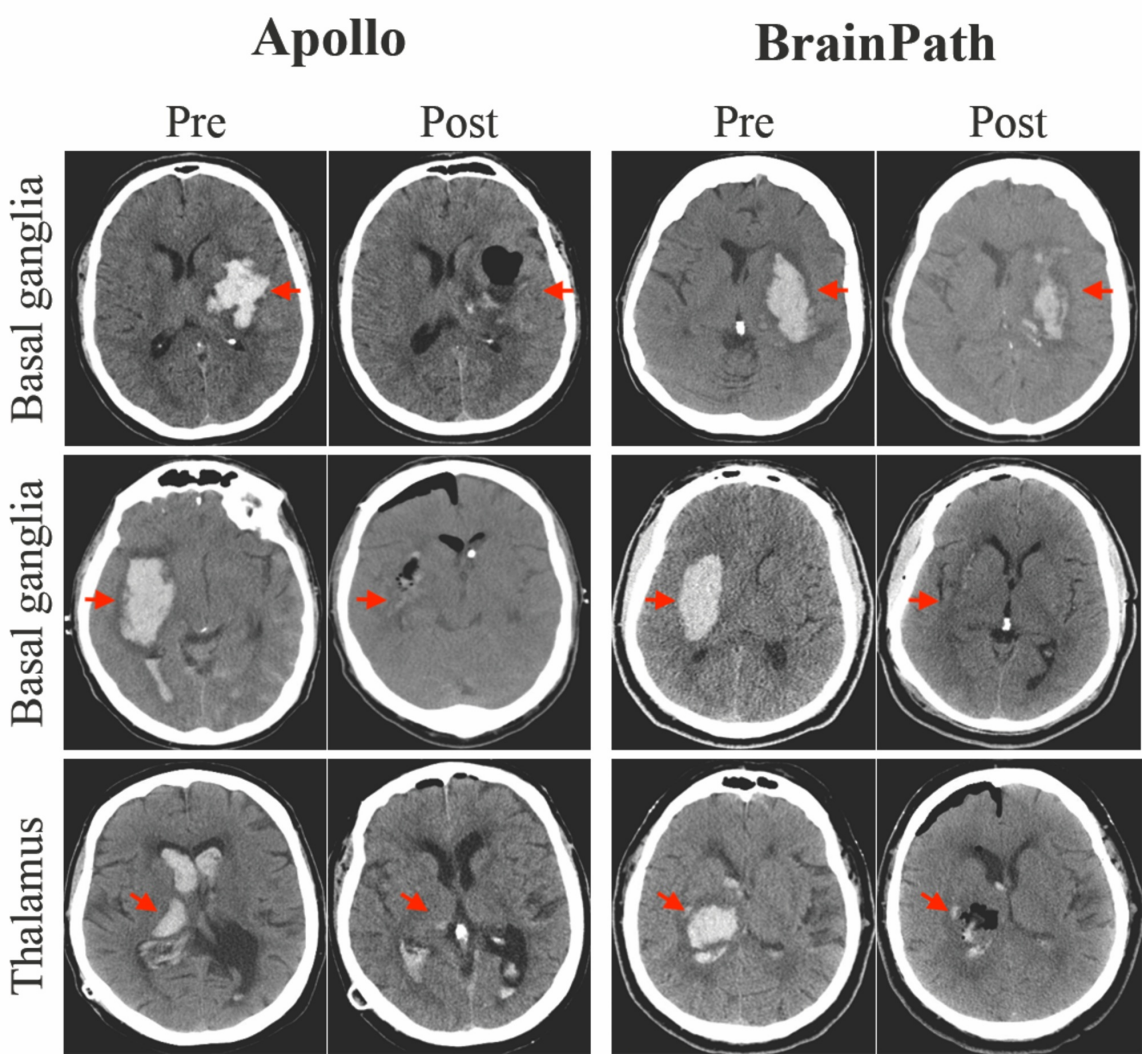
Pre- and postoperative axial CT images of the head of matched left basal ganglia, right basal ganglia, and right thalamic intracranial hemorrhages treated with the Apollo and BrainPath systems CT: Computed Tomography Apollo (Penumbra Inc, Alameda, California, US); Nico BrainPath (Indianapolis, Indiana, US)

Based on age, gender, hematoma location and laterality, and volume, five cases performed with Apollo were matched to cases done with the BrainPath system (Figure 2).

Patient characteristics

The median age in both groups was 61 years with a male to female ratio of 1.5 to 1. Locations included basal ganglia, thalamus, intraventricular, and lobar in the frontal and frontotemporal regions. The mean hematoma volume in the Apollo and BrainPath groups was 50.7 and 42.3 ml, respectively. The median preoperative GCS score and ICH score were 9 and 10 and 3 and 2, respectively [7]. Median time from onset to evacuation was two days in both groups. The mean distance from the brain surface to the clot was 41.8 and 43.2 mm, respectively. There were no statistical differences between groups for characteristics not used for matching (Table 1).

**Table 1:** Patient characteristics and outcome measures GCS: Glasgow Coma Scale; ICH: Intracranial Hemorrhage; IVH: Intraventricular Hemorrhage; SD: Standard Deviation; PEG: Percutaneous Endoscopic Gastrostomy; mRS: Modified Rankin Score ^1^One BrainPath patient was lost to follow-up. *Pearson test Apollo (Penumbra Inc, Alameda, California, US); BrainPath (Indianapolis, Indiana, US)

Parameter	Apollo	BrainPath	P-value
Number of patients	5	5	
Age (years; median, range)	61 (38-71)	61 (45-80)	0.49
Gender			
Male	3 (60%)	3 (60%)	0.55*
Female	2 (40%)	2 (40%)	
GCS on admission (median, range)	9 (3-11)	10 (7-14)	0.2
ICH score	3 (1-4)	2 (1-3)	0.55
Location			
Basal ganglia	2 (40%)	2 (40%)	0.6*
Thalamus	1 (20%)	1 (20%)	
IVH	1 (20%)	1 (20%)	
Frontal/Frontotemporal	1 (20%)	1 (20%)	
Associated IVH	3 (60%)	2 (40%)	0.3
Laterality			
Left	2 (40%)	2 (40%)	0.81*
Right	2 (40%)	2 (40%)	
Bilateral	1 (20%)	1 (20%)	
Volume (ml; mean ± SD)	50.7 ±23.9	42.3 ± 9.1	0.5
Brain surface to clot (mm; mean ± SD)	41.8 ±12.2	43.2 ± 7.9	0.83
Etiology			
Hypertension	3 (60%)	4 (80%)	0.29*
Symptomatic aneurysm	2 (40%)	1 (20%)	
Time from onset to evacuation (days; median, range)	2 (1-4)	2 (0-3)	0.52
Postoperative volume (ml; mean ± SD)	13.2 ±12.4	11.9 ± 10	0.86
Tracheostomy/PEG tube	3 (60%)	3 (60%)	1
GCS upon discharge (median, range)	10 (3-15)	3 (3-15)	0.56
Length of stay (days; median, range)	16 (9-34)	19 (3-58)	0.82
Length of follow-up^1^ (days; median, range)	55 (34-258)	21 (10-713)	0.34
mRS on last follow-up^1^			
0-2	0	1 (25%)	0.27*
3-5	2 (40%)	1 (25%)	
6	3 (60%)	2 (50%)	

Outcome measures

There was a significant reduction in the hematoma volume with evacuation using either system. The mean postoperative hematoma volume was 13.2 and 11.9 ml in the Apollo and BrainPath groups, respectively. The median GCS at discharge and length of stay were 10 and 3 and 16 and 19 days, respectively. No operative complications occurred in any of the cases. There were no differences in mRS at follow-up between the two groups after the median follow-up duration of 55 and 21 days, respectively (range 10 days to two years). There were four mortalities, two in each group, at last follow-up. Notably, one patient in the Apollo group who required ventriculoperitoneal shunting sustained a fall and died from a subdural hematoma months later. One patient who underwent BrainPath evacuation developed acute disseminated encephalomyelitis in the postoperative period and remained severely disabled (Table 1).

## Discussion

The current study is the first to compare image-guided, minimally invasive hematoma evacuation comparing the Apollo system and the BrainPath approach in a series of consecutive cases utilizing both modalities. Albeit the smallness of the sample size, five cases of each technique were matched based on patient age, gender, hematoma location and laterality, as well as volume. Both systems were found to be safe and achieved comparable clot evacuation without any surgical complications. Despite a significant reduction in hematoma burden and impressive improvement on imaging after evacuation, functional outcomes were disappointing, though no worse than the natural history. Spontaneous ICH is associated with the highest mortality among all stroke types [1]. In the present study, the majority of patients were dead or dependent at last follow-up. The median follow-up length, however, falls short of the six-month follow-up hypothesized to be necessary to prove a neurological benefit.

Pathophysiologic process surrounding nontraumatic intracerebral hemorrhage

Indeed, nontraumatic ICH, or hemorrhagic stroke, has the highest mortality rate among the different stroke types at 62% at one year [1]. Approximately 80%-90% of all ICH cases are classified as primary without underlying cerebrovascular or other pathologic abnormality and result from the rupture of a small artery damaged by chronic hypertension or amyloid angiopathy. Secondary ICH is much less common and a consequence of cerebrovascular or other pathologies, such as brain aneurysms or brain arteriovenous malformations, hemorrhagic tumors, or coagulopathy. Injury from ICH is characterized by a primary and a secondary phase. The primary insult stems from mechanical injury and ischemia of the surrounding brain structures, leading to cytotoxic edema and necrosis [6]. The release of blood products and proteins from the hematoma initiates the phase of secondary damage characterized by vasogenic edema from blood-brain barrier breakdown, predominantly necrotic, but also apoptotic cell death, and may persist up to two weeks.

The role of surgical intracerebral hemorrhage evacuation

Evacuation of the ICH aims at the reduction in mass effect and limitation of pathologic interactions between the hematoma and healthy perihematoma tissue. With the exception of cerebellar ICH, the usefulness of surgical evacuation of supratentorial ICH is not well established [8]. While failure of the Surgical Trial for Intracerebral Hemorrhage (STICH) I and II trials [2-3] has been attributed to a number of factors such as a high crossover rate to surgical intervention [6,8], the fundamental flaw may have been the invasive nature of the traditional, open surgical approach itself. Current hopes for surgical clot evacuation to prove beneficial in the management of ICH lie on minimally invasive techniques [9-12]. Despite recent efforts, this concept is not entirely new. In 1989 Auer et al. published a randomized study of 100 patients with supratentorial subcortical ICH comparing endoscopic surgery and medical management. For subcortically located ICH, endoscopic evacuation resulted in lower mortality and a higher percentage of good functional. This benefit was not observed for basal ganglia and thalamic locations [13]. Recently, the MISTIE phase II trial, designed to explore image-guided catheter aspiration and delivery of tPA in patients with ICH volume greater or equal 20 mL after demonstration of clot stability for six hours, proved safety and a potential advantage of improved functional outcome (mRS 0-3) at six months in the interventional arm [4]. The follow-up study MISTIE III, a 500-patient trial with the goal to demonstrate an improvement in functional outcome using the MISTIE technique, is well underway and results are expected to be reported soon. The results of the MISTIE III study will likely determine the fate of minimally invasive evacuation of ICH, regardless of the technique. Whereas clot evacuation in MISTIE is performed without direct visualization and is largely passive after the initial aspiration, other image-guided, minimally invasive clot evacuation strategies involve direct clot visualization using endo- or exoscopes and active clot removal. These include the endoscope-assisted Apollo and exoscope-assisted BrainPath systems. 

Apollo and BrainPath intracerebral hemorrhage evacuation

Currently, the literature on either one of these image-guided, minimally invasive approaches is restricted to single-arm case series [5-6,14-16]. Labib et al. reported 39 patients presenting with ICH that underwent BrainPath evacuation [6]. Median GCS was 10 and the basal ganglia and thalamus regions were involved in 46% of the cases. The median hematoma volume of 36 ml and depth of 1.4 centimeters were notably lower than in the present study and may be an explanation of the vast differences in outcome. Whereas the majority of patients in the present study were dead or dependent, Labib et al. reported a significant improvement in GCS from admission to discharge along with a rate of functional independence of 52% (mRS ≤ 2) and no mortalities [6]. The largest series using the Apollo system included 29 patients. The mean preoperative hematoma volume was comparable to the present study at 45 milliliters. Mortality was 13.8% and only 6.9% of patients had been discharged home at study termination, indicating a low rate of functional independence [15]. There are two ongoing randomized controlled trials evaluating these two techniques. The Early MiNimally-invasive Removal of IntraCerebral Hemorrhage (ENRICH) trial evaluates the Brainpath technique and has, as of October 2018, enrolled approximately half of the anticipated 300 subjects [17]. The Minimally Invasive Neuro Evacuation Device (MIND) trial has recently started enrollment to assess endoscopic ICH evacuation using Artemis (Penumbra, Alameda, CA, US), the next generation aspiration device following Apollo, with an anticipated enrollment of 500 participants [18].

Limitations

The retrospective character and small sample size introduce bias and limit the ability to perform a robust statistical analysis. No convincing conclusions regarding functional outcome can be made in such a small cohort. The decision to use the Apollo versus the BrainPath system was at the discretion of the treating neurosurgeon. No unified criteria on indications for image-guided, minimally invasive ICH evacuation had been determined.

## Conclusions

In the present, small, matched cohort study, both the Apollo and BrainPath techniques are safe and achieved satisfactory clot evacuation. Nevertheless, the functional outcome in this patient population remains poor in the majority of cases and the selection of a subgroup of patients with ICH who will benefit from evacuation with either modality remains elusive. We anticipate ongoing randomized controlled trials, such as ENRICH and MIND, will demonstrate the safety and efficacy of these minimally invasive approaches to ICH.
